# From Local Action to Global Policy: A Comparative Policy Content Analysis of National Policies to Address Musculoskeletal Health to Inform Global Policy Development

**DOI:** 10.34172/ijhpm.2022.7031

**Published:** 2023-01-04

**Authors:** Carmen Huckel Schneider, Sarika Parambath, James J. Young, Swatee Jain, Helen Slater, Saurab Sharma, Deborah Kopansky-Giles, Lyn March, Andrew M. Briggs

**Affiliations:** ^1^Menzies Centre for Health Policy and Economics, Faculty of Medicine and Health, University of Sydney, Sydney, NSW, Australia.; ^2^Center for Muscle and Joint Health, Faculty of Health Sciences, University of Southern Denmark, Odense, Denmark.; ^3^Department of Research, Canadian Memorial Chiropractic College, Toronto, ON, Canada.; ^4^Sydney Musculoskeletal, Bone & Joint Health Alliance, Faculty of Medicine and Health, University of Sydney, Sydney, NSW, Australia.; ^5^Curtin School of Allied Health, and Curtin enAble Institute, Faculty of Health Sciences, Curtin University, Perth, WA, Australia.; ^6^School of Health Sciences, Faculty of Medicine and Health, University of New South Wales, Sydney, NSW, Australia.; ^7^Centre for Pain IMPACT, Neuroscience Research Australia, Sydney, NSW, Australia.; ^8^Department of Family & Community Medicine, University of Toronto, Toronto, ON, Canada.; ^9^Florance and Cope Professorial Department of Rheumatology, Royal North Shore Hospital and Kolling Institute, University of Sydney, Sydney, NSW, Australia.

**Keywords:** Policy Content Analysis, Global Policy, Musculoskeletal Health, Policy Learning

## Abstract

**Background:** Global policy to guide action on musculoskeletal (MSK) health is in a nascent phase. Lagging behind other non-communicable diseases (NCDs) there is currently little global policy to assist governments to develop national approaches to MSK health. Considering the importance of comparison and learning for global policy development, we aimed to perform a comparative analysis of national MSK policies to identify areas of innovation and draw common themes and principles that could guide MSK health policy.

**Methods:** Multi-modal search strategy incorporating a systematic online search targeted at the 30 most populated nations; a call to networked experts; a specified question in a related eDelphi questionnaire; and snowballing methods. Extracted data were organised using an *a priori* framework adapted from the World Health Organization (WHO) Building Blocks and further inductive coding. Subsequently, texts were open coded and thematically analysed to derive specific sub-themes and principles underlying texts within each theme, serving as abstracted, transferable concepts for future global policy.

**Results:** The search yielded 165 documents with 41 retained after removal of duplicates and exclusions. Only three documents were comprehensive national strategies addressing MSK health. The most common conditions addressed in the documents were pain (non-cancer), low back pain, occupational health, inflammatory conditions, and osteoarthritis. Across eight categories, we derived 47 sub-themes with transferable principles that could guide global policy for: service delivery; workforce; medicines and technologies; financing; data and information systems; leadership and governance; citizens, consumers and communities; and research and innovation.

**Conclusion:** There are few examples of national strategic policy to address MSK health; however, many countries are moving towards this by documenting the burden of disease and developing policies for MSK services. This review found a breadth of principles that can add to this existing work and may be adopted to develop comprehensive system-wide MSK health approaches at national and global levels.

## Background

**Key Messages**

** Implications for policy makers**

- Despite a high burden of disease, very few countries have a national strategy/policy to specifically address musculoskeletal (MSK) health.
- The development of such national-level policies is hindered by a lack of global policy, technical guidance and health performance indicators that prioritise premature mortality over long-term morbidity.
- This study has drawn together national and regional level policies addressing a range of MSK conditions to determine key themes and underlying principles to inform the foundations of global policy and advocate for its development.
- These principles can also be transferred and localised to underpin the development of health systems strengthening responses to address MSK health.

** Implications for the public**

It is known that musculoskeletal (MSK) disorders, including conditions such as arthritis, fragility fractures, low back pain and neck pain are the leading cause of disability worldwide. The complex nature of these conditions requires coordinated models of care that include prevention, treatment and management. In this study we found that many countries are at the early stages of developing strategies to address these disorders or have no strategy at all. This study combined the learnings from many countries about the way they develop strategies and policies to assist in improving services to address these disorders. We then developed a series of content areas and principles that can be used to check, strengthen and monitor health systems to improve MSK health globally.

Musculoskeletal (MSK) health refers to the integrity and function of the locomotor system, comprising muscles, bones, joints, nerves and associated connective tissues. MSK health is required for mobility, physical function, and dexterity across the life course. Impairments of the MSK system are typically associated with pain, reduced physical function and mobility, often leading to reduced social engagement (eg, work, schooling) and quality of life. Encompassing a suite of more than 150 disorders MSK impairments comprise MSK conditions (eg, arthritis, osteoporosis) pain manifesting in MSK structures (eg, low back pain) and MSK injury and trauma (eg, fractures).^1^ Despite MSK health conditions being the leading cause of disability worldwide representing 17% of the global years lived with disability in 2019, global strategy and policy guidance lags behind that for other non-communicable diseases (NCDs), particularly in low resource settings.^2-4^ Global policy to guide action on MSK health is in a nascent phase. There is currently little global policy or strategic direction that serves as technical guidance to assist national governments in developing whole-of-system approaches to MSK health.^5,6^

 Global policy serves to inform best-practice targets and provide guidance on the technical implementation of policies at local, national and regional levels. The development of global policy, however, evolves through a long process of agenda-setting, policy development, debate, legitimation and dissemination, with each stage entailing political, procedural and technical aspects.^6,7^ The goal is the facilitation of policy learning; where unique experiences and local knowledge are observed, combined and analysed to provide a basis for globally-relevant learnings. The challenge is creating global policy that is grounded in local experience but interpreted into principles that find relevance and applicability across contexts. In recent years these development processes have been helpful for rapidly progressing global policy with respect to mental health,^8^ NCDs,^9^ antimicrobial resistance,^10^ tobacco control and alcohol and other drugs.^9,11-13^

 Despite the attributed high health and economic burden, MSK health receives little attention on the global stage, and has not featured prominently in recent global goal setting and consequently national policies on NCDs.^6^ Despite overwhelming and increasing recognition of the burden of MSK disorders,^14^ recent research has identified gaps in the evidence base in terms of effective policy solutions.^15^ The purpose of this review is to extend this knowledge base and contribute to the feasibility and promise of global MSK policy. In acknowledging that the development of global policy is grounded in phases of policy comparison and learning from local experiences, we aimed to, (1) perform a comparative analysis of national MSK policies and develop a snapshot of current national MSK policy approaches and priorities; (2) collate themes covered from the combined content of existing national policies; and (3) draw key principles from the existing pool of local policies that can be used in policy learning.

 This study was undertaken as part of a three-phase project that included key informant interviews with MSK policy experts and a global eDelphi to determine prioritised components for a global strategy for improving MSK health.^16-18^ The goal of this paper is to provide the detailed findings from our policy analysis to assist policy-makers at both national and international levels. We also aim to outline a detailed method for undertaking comparative policy content analysis for this purpose, in the hope that it can be useful for facilitating global policy learning for other vital health challenges.

## Methods

###  Design

 We undertook a systematic comparative policy content analysis of MSK heath policy documents adapted from the framework proposed by Arksey and O’Malley,^19^ and used by Anderson et al,^20^ for policy mapping.

###  Inclusion Criteria: Document Types

 Documents were included where they described national, sub-national or multi-national level health policies for a MSK health condition(s) that were government-issued – or co-sponsored with government – labelled as ‘policy,’ ‘strategy,’ ‘framework,’ ‘action plan’ or similar, consistent with definitions used previously.^3^ Policies needed to focus on MSK health generally, MSK pain, or any specific MSK condition.

###  Document Collection

 We conducted a broad search for national MSK policies via four search methods.

 First, we performed a systematic online desktop search for national MSK policies and guidelines from the 30 most populous countries, informed by United Nations World Population Prospects 2019. A comprehensive search in English was performed for each country using Google as the search engine from July 1, 2020 to August 15, 2020. Authors JJY, SM, and SP used a combination of search terms (policy OR strategy OR action plan OR strategic framework OR health indicators) AND (musculoskeletal OR chronic pain OR rheumatology OR orthopedics OR pediatrics OR rehabilitation OR gerontology/geriatrics OR physiotherapy OR chiropractic) along with the country name, to locate potential national policy documents. See Supplementary file 1 for full search terms. When search terms led to the webpage of a national government agency relevant to MSK health or chronic pain, manual searching of the entire website for relevant documents was performed. In addition, manual searching of other linked government agencies webpages was also performed. Further, we made contact with experts in MSK health with language and contextual knowledge of included countries. Contacts from countries not included in the top 30 most populous nations were also asked to identify any potentially relevant documents.

 Second, we identified policy documents via requests to the Global Alliance for Musculoskeletal Health International Coordinating Council members and policy researchers (expert round), including those with access to raw data from an earlier integrated NCD policy review of OECD (Organisation for Economic Co-operation and Development) member states.^3^ The request was deliberately broad to enable the collation of policies that could fit any of the potential criteria for inclusion.

 Third, we extracted data from a related project that used an eDelphi method to collect information from the global MSK community on systems strengthening opportunities and priorities.^17^ As part of the eDelphi, panel members were asked to identify national policies for MSK health in their country of residence. The question was posed in an eDelphi survey administered between October 2 and November 8, 2020. Of the 674 respondents to the original eDelphi, which included stakeholders with a policy, clinical, health services or lived experience lens, 22 answered this purposive question, providing links to policy documents. This step represented a secondary data analysis of an existing dataset for which Human Research Ethics Committee approval had been granted by Curtin University, Australia, in 2020 (HRE2020-0183).

 Fourth, we examined all documents for reference to additional MSK policy documents following snowballing methods. All documents that were not originally in English or German were translated using an online document translator (https://www.onlinedoctranslator.com/en/) as previously.^3^

###  Inclusion Criteria (Document Content) and Document Selection

 An initial review of documents included in the pool showed a wide variety of document types, publishers, purposes and formats, with only three specifically designed as national-level policies for MSK health. Inclusion criteria outlined above were further refined to ensure the analysis was targeted at the study aims of comparing national MSK health policies, but also included documents that target MSK specific conditions. Our final inclusion criteria were: policy documents that are (1) government-issued; published by official government departments, or explicitly endorsed by government departments as representing the policy of a specified jurisdiction; (2) targeted at the population-level improvement in MSK health; or containing a substantial sections/chapters dedicated to MSK health (general) or any of the following clinically meaningful categories: non-cancer pain conditions (either regional or widespread); osteoarthritis; injury (including occupational), excluding trauma; and auto-immune inflammatory conditions with MSK manifestations; (3) was the current version (if regularly updated) with a publication/coverage date not older than 2010; and (4) contains jurisdiction-wide strategies, action plans or system-level models of care. We did not disaggregate classification categories for MSK conditions based on International Classification of Diseases 11th Revision (ICD-11), since many policies pre-dated this classification system and did not describe conditions at the granular level of ICD classification. Rather, we took a pragmatic approach to meaningfully group disease/condition classifications that aligned with classification approaches used across the policies. We defined a system-level model of care as a document including a care pathway that includes prevention, diagnosis, treatment, rehabilitation and recovery, and identified the roles played by different providers within the pathways, their responsibilities, and information on how the different providers connect within the system. Documents that were primarily targeting related aspects of health, such as NCDs, population health, occupational health and injuries were only included if they contained a substantial component addressing the above criteria.

 Authors CHS and SP performed a review of documents for inclusion/exclusion, with verification of those meeting inclusion criteria undertaken by JJY and AMB. All excluded documents were reassessed independently, with discordance resolved through consensus meetings.

###  Data Analysis

 Authors SP and CHS collated and prepared all documents for extraction. Two data extraction templates were developed in Excel, one for descriptive level information about the documents and a second for the comparative content analysis and abstraction of principles. Spreadsheets were used for a comparative policy content analysis, undertaken in three steps: (1) categorisation, (2) descriptive thematic analysis, and (3) interpretative analysis.

####  Categorisation

 Authors CHS and SP read all policy documents independently and extracted high-level data about each document including, country of origin, jurisdiction, publisher, date, and target condition(s). We then identified and extracted the overarching targets, goals and objectives specified in each policy document and inductively open-coded these texts to identify themes. Open coding was undertaken manually by extracting excerpts for collation using Excel, with regularly review, consolidation and merging.

####  Analysis

####  Descriptive Thematic Analysis

 We identified analysis categories *a priori*, initially based on World Health Organization (WHO) Building Block elements, given the universal acceptance and familiarity of this model with policy-makers addressing health systems strengthening. We assessed the construct validity and ‘fit’ of this model through a round of inductive coding of 6 policy documents from the yield that appeared in first order through the original search. Following this inductive coding, we added two further categories to the WHO Building Blocks, including “Citizens, Consumers and Communities” and “Research and Innovation.” These categories aligned intuitively with the themes identified in our earlier qualitative research which formed the framework for the ‘Empirically derived logic model for a global strategy for MSK health.’^17^ These categories were used to deductively code the remainder of the texts. CHS and SP were each allocated different analysis categories and extracted texts that were open-coded to determine the number and type of sub-themes included in the texts within each category. Definitional boundaries around each sub-theme were then agreed upon, in conference between SP and CHS, and reported for discussion to all authors. Sub-themes were refined, expanded or merged as necessary.

####  Interpretive Analysis

 To extract key principles underlying the meaning of texts, we read, discussed and interpreted underlying meanings from across multiple texts within each category, reflecting on position, overarching goals, common linguistic patterns and purpose of texts.^17^ Principles were developed to represent the common underlying purpose of a policy action for achieving policy goals. Abstracted principles were presented at working group and project group meetings and discussed with all authors and refined for internal validity and consistency purposes.

## Results

###  Overview of Included Policies

 From an initial yield of 165 documents, 6 were excluded as duplicates. A further 7 documents were excluded as falling outside of the specified date range and 111 documents did not meet the criteria of being a policy document or did not have a substantial MSK component. It is notable that while our search was not designed to capture clinical and treatment guidelines, or reports on MSK burden and risk factors, we identified a far greater number of these than we did policy documents in our final yield. These peripheral documents also came from a greater range of high-, low- and middle-income countries (LMICs) (see Table 1) than policy documents. After exclusions, 41 (24.8%) policy documents remained for analysis, representing 22 countries including Australia,^21-23^ Belgium,^24^ Canada,^25-27^ Chile,^28^ Columbia,^29^ Denmark,^30^ Finland,^31^ France,^32,33^ Hungary,^34^ Italy,^35^ Ireland,^36^ New Zealand,^37-39^ Norway,^40,41^ Portugal,^42^ Republic of Korea,^43^ Spain,^44^ Switzerland,^45,46^ Turkey,^47^ the United Kingdom (England),^48-50^ the United Kingdom (Scotland),^51,52^ the United Kingdom (Wales),^53^ the United States^54-58^; and two multi-national regions (European Union^59,60^; international^61^).We did not identify any eligible documents from LMICs, however, a number of MSK-relevant documents from LMICs were identified from the search (see Table 1). Of the 118 documents excluded after removal of duplicates, most were classified as clinical guidelines (n = 56), government reports on burden of disease (n = 14), non-governmental calls to action and reports (n = 17), and other non-policy literature (n = 14).

**Table 1:** Characteristics of Policy Documents

**Document Number; Country**	**Title (Publisher)**	**Year Published (Years Operational)**	**Scope or Goal of the Policy**
Australia 1^22^	Australian National Strategic Action Plan on Arthritis (Australian Department of Health)	2019	Coordinate a national response to arthritis across state and territory governments, health service providers and funders, clinicians, consumers, researchers and research funders.
Australia 2^23^	Australian National Strategic Action Plan on Osteoporosis (Australian Department of Health)	2019	Address the urgent need for a national strategic response to the increasingly complex challenges and burden of osteoporosis across Australia.
Australia 3^21^	Australian National Strategic Action Plan for Pain Management (PainAustralia/Australian Department of Health)	2019	Improve the quality of life for people living with pain and the pain burden for individuals and the community is minimised.
Belgium 1^24^	Management of chronic pain in Belgium: past, present and future (Federal Public Agency for Public Health, Safety, Food and the Environment, Belgium)	2011	Mapping the general situation of the problem of chronic pain, description of the current Belgian situation and finally recommendations for future changes.
Canada 1^26^	Institute of Musculoskeletal Health and Arthritis Strategic Plan 2014-2018: Enhancing Musculoskeletal, Skin and Oral Health (Canadian Institute of Health Research)	2014 (2014-2018)	Outline strategic investment priorities for the period 2014-2018 of the Institute of Musculoskeletal Health and Arthritis.
Canada 2^25^	Joint Action on Arthritis - a framework to improve arthritis prevention and care in Canada (Arthritis Alliance of Canada)	2012	Galvanize action around long-term strategies to improve arthritis prevention, and quality and efficiency of care. Facilitate and focus collaboration among governments and arthritis stakeholders in awareness, models of care and research.
Canada 3^27^	Chronic pain in Canada: laying a foundation for action (Health Canada)	2019	Improving the lives of all of Canada's people and making this country's population among the healthiest in the world as measured by longevity, lifestyle and effective use of the public healthcare system.
Chile 1^28^	National Health Strategy to complete the Health Objectives of the Decade (Government of Chile)	2011 (2011-2020)	To reduce the impact of chronic communicable and NCD.
Columbia 1^29^	Plan Nacional de Seguridad y Salud en el Trabajo 2013-2021 (National Plan for Safety and Health at Work 2013-2021) (Ministry of Labor)	2014 (2013-2021)	Promotion of the transversality of safety and health at work in public policies; strengthening safety and health at work; strengthening the promotion of safety and health of workers and the prevention of occupational risks, and optimisation and guarantee of the recognition of worker benefits in the general labour risk system.
Denmark 1^30^	Recommendations for multidisciplinary management of low back pain (National Health Board of Denmark)	2017	Increase the quality of assessment, treatment and prevention and rehabilitation services as well as in follow-up of the course about people with chronic low back pain.
European Union 1^60^	European action towards better musculoskeletal health (EFORT/EULAR/IOF)	2017	To prevent MSK problems and conditions where possible, and to ensure that those people with MSK conditions enjoy a life of fair quality as independently as possible.
European Union 2^59^	Occupational health and safety risks in the healthcare sector-Guide to prevention and good practice (European Commission)	2010	Better, healthier and more competitive workplaces and a corporate culture where managers and workers (as experts on their workplaces) discuss work processes together in a continuous improvement process including all related risks and possible measures for improvements.
Finland 1^31^	National Action Plan for the Treatment of Chronic Pain and Cancer Pain (Ministry of Social Affairs and Health, Finland)	2017	Develop a national plan for the treatment of chronic and cancer pain to build a multidisciplinary and multidisciplinary model of care from primary care to specialist care.
France 1^32^	Monitoring Plan for National Pain Program (Ministry of Health and Solidarity, France)	2006 (2006-2010)	To continue and strengthen the policy initiated for more than 10 years to improve pain management.
France 2^33^	Occupational Health Plan 2016-2020 (Ministry of Labour, France)	2016 (2016-2020)	Outline objectives and actions in relations to primary prevention and culture of prevention; and quality of life at work, job retention and performance.
Hungary 1^34^	Health Hungary 2014-2020- Health Sector Strategy (Ministry of Human Resources, State Secretariat for Health, Government of Hungary)	2015	A whole-of-system approach to improving health outcomes for Hungarian citizens and the efficiency of the system. Health awareness among citizens to act proactively; the importance of industry and government in collaboration in order to make the country healthier and live a more health-conscious life.
International 1^61^	A framework to evaluate musculoskeletal models of care (Global Alliance for Musculoskeletal Health of the Bone and Joint Decade)	2016	Improving consumer health outcomes and system-relevant outcomes for NCDs, particularly MSK conditions.
Italy 1^35^	National Plan for Chronic Disease (Directorate-General of Health Programming, Italy)	2016	Contribute to the improvement of protection for people suffering from chronic diseases, reducing the burden on the individual, family and the social context. Improving the quality of life and making health services more effective and efficient in terms of prevention, support and ensuring greater uniformity and equity of access.
Ireland 1^36^	The model of care for rheumatology in Ireland (Royal College of Physicians of Ireland)	2015	Develop a chronic disease model of care to facilitate the right person, right place, first-time approach to patients with rheumatic and MSK disorders.
New Zealand 1^37^	National Health Committee low back pain: a pathway to prioritisation (National Health Committee)	2014	Explore the current approach to care for patients with low back pain and identify interventions where the National Health Committee could conduct a further evaluation in order to improve health outcomes and efficiency within the New Zealand health system.
New Zealand 2^38^	Low Back Pain (LBP) Tier 2 Assessment (National Health Committee)	2015	Analyse the current delivery of chronic low back pain services, the evidence for effective interventions and proposes the development of a complete model of care for chronic low back pain.
New Zealand 3^39^	The Mobility Action Program (New Zealand Ministry of Health)	2015	Ensure that the right care is provided in the right place at the right time by practitioners with the right resources.
Norway 1^40^	Norway: Together for a good working environment (European Agency for Safety and Health at Work)	2007-2010	Report on tripartite cooperation between the government and social partners, represented by trade unions and employers’ organisations in focusing on improving the work environment, reducing sick leave and increasing the retirement age.
Norway 2^41^	Public Health Report 2018-2019: Good Life in a Safe Society (Norwegian Ministry of Health and Care Services, Government of Norway)	2018 (2018-2019)	To reduce the incidence of premature death and ill health due to NCDs, in line with the global goals of the WHO.
Portugal 1^42^	National Strategic Plan for Pain Prevention and Control (Directorate General Health, Portugal)	2017	Reduce the prevalence of uncontrolled pain in the Portuguese population; improve the quality of life of patients with pain; and rationalize resources and control the costs necessary for pain control.
Republic of Korea 1^43^	The 3rd National Health Promotion Plan 2011-2020 (Korean Ministry of Health and Welfare)	2011	To create a healthy community through increasing life expectancy and achieving health equity.
Spain 1^44^	Pain Care Strategy 2017-2020 (City of Madrid)	2017 (2017-2020)	Provide adequate assistance to all patients with pain who are cared for in the health facilities of the Madrid Health Service.
Switzerland 1^45^	National strategy for the prevention of noncommunicable diseases 2017-2024 (Swiss Confederation, Federal Department of Home Affairs, Federal Office of Public Health).	2017 (2017-2024)	More people stay healthy or have a high quality of life despite chronic illness. Fewer people suffer from avoidable NCDs or die prematurely. Regardless of their socioeconomic status, people are empowered in their efforts to cultivate a healthy lifestyle in a health-promoting environment.
Switzerland 2^46^	National Strategy for Musculoskeletal Disorders 2017-2022 (Rheumaliga Schweiz)	2017 (2017-2022)	Guarantee the strategic orientation of activities in the areas of early detection and prevention of risk factors (secondary prevention) as well as in the care and treatment of those affected (tertiary prevention).
Turkey 1^47^	Turkey Musculoskeletal Disease Prevention and Control Program 2015-2020 (Ministry of Health, Turkey)	2015 (2015-2020)	Plan activities for the prevention, early diagnosis and treatment of the MSK diseases within the scope of the prevention and control program; take permanent and effective steps for their early diagnosis and treatment; reduce rehabilitation services and disability and ensure that individuals benefit from the highest attainable health standard suitable for their own needs.
United Kingdom (England) 1^48^	Developing partnerships and a whole-system approach for the prevention of musculoskeletal conditions in England (Public Health England)	2018	Shared narrative and journey for a public health approach to MSK health.
United Kingdom (England) 2^50^	Musculoskeletal core capabilities framework for the first point of contact practitioners (Health Education England and NHS England)	2018	Ensure a person-centred approach in the first stages of managing any MSK problem with which a person may present.
United Kingdom (England) 3^49^	Musculoskeletal health: A 5-year strategic framework for prevention across the life course (Department of Health and Social care, Public Health England and Department for Work and Pensions)	2019 (2019-2023)	To promote MSK health and to prevent MSK conditions.
United Kingdom (Scotland) 1^52^	Allied health professional (AHP) musculoskeletal pathway framework (National Minimum Standard) (The Scottish Government)	2014	Ensure that people requiring MSK services, receive the quality of care and the support they require, at the appropriate time by the appropriate person.
United Kingdom (Scotland) 2^51^	Future provision of specialist residential chronic pain management services in Scotland: consultation report (Scottish Government)	2014	Respond to the findings of the consultation as set out in the independent report ‘*The Provision of Residential Chronic Pain Services in Scotland: Analysis of Consultation Responses*’ and sets out the Scottish Government’s proposals for taking forward the development of these services.
United Kingdom (Wales) 1^53^	Living with persistent pain in Wales (Welsh Government)	2019	Provide advice to those experiencing persistent pain and their families, and health and social care professionals; taking a patient-centred approach.
United States of America 1^58^	Improving pain management and support for workers with musculoskeletal disorders: policies to prevent work disability and job loss (US Department of Labor/IMPAQ International)	2017	Promote an effective, evidence-based, and timely approach to managing MSK pain and disability that applies a biopsychosocial treatment model to restore workplace function and prevent or minimize work disability as early as possible.
United States of America 2^56^	National Institute for Occupational Safety and Health Musculoskeletal Health Program (National Institute for Occupational Safety and Health)	2019	Reduce work-related MSK disorders such as carpal tunnel syndrome and low back pain.
United States of America 3^57^	A National Public Health Agenda for Osteoarthritis: 2020 Update (Osteoarthritis Action Alliance, Centre for Disease Control and Prevention, Arthritis Foundation)	2020	Adults with osteoarthritis are able to live full lives with less pain, stiffness, and disability; greater mobility; and preserved function and independence.
United States of America 4^55^	National Pain Strategy: A Comprehensive Population Health-Level Strategy for Pain (Department of Health and Human Services/Interagency Pain Research Coordinating Committee)	2011	Decrease the prevalence of pain across its continuum from acute to high-impact chronic pain and its associated morbidity and disability across the lifespan. Reduce the burden of pain for individuals, their families, and society as a whole.
United States of America 5^54^	Relieving Pain in America: A Blueprint for Transforming Prevention, Care, Education, and Research (Institute of Medicine)	2011	Transform the way pain is understood, assessed, treated, and prevented. Improve the care of people who experience pain, the training of clinicians who treat them, and the collection of data on pain in the United States.

Abbreviations: MSK, musculoskeletal; WHO, World Health Organization; NCD, non-communicable disease; EULAR, European League Against Rheumatism; EFORT, The European Federation of National Associations of Orthopaedics and Traumatology; NHS, National Health Service; IOF, International Osteoporosis Foundation.

 Policies collated via each method, are summarized in a Preferred Reporting Items for Systematic Reviews and Meta-Analyses (PRISMA)-aligned flow chart (Figure). We included countries of origin of excluded documents in this figure to demonstrate the breadth of countries that are recognising and working towards action on MSK health, even where policies are not yet developed.

**Figure:**
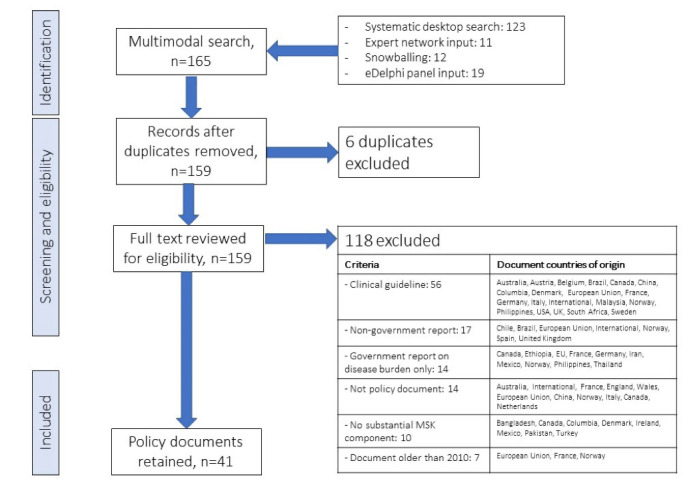
Comparative Policy Content Analysis of National Polices to Address Musculoskeletal Health: PRIMA Diagram. Abbreviation: PRISMA, Preferred Reporting Items for Systematic Reviews and Meta-Analyses.

###  Policy Characteristics

 The documents we analysed varied greatly in form and purpose (see Table 2). Documents ranged from book-length reviews of the current health system, MSK services disease burden and future reform initiatives (for example, documents USA 1 and Belgium 1), to stand-alone tables of goals, target roles and responsibilities but without guiding text (for example, document Columbia 1).

**Table 2:** Weighting of Musculoskeletal Conditions Found in Policy Documents

**Document**	**MSK Conditions**							
	**MSK General**	**Non-cancer Pain**	**Low Back Pain**	**Osteoarthritis **	**Inflammatory Conditions**	**Occupational Health and Injury**	**Osteoporosis and Fragility Fractures**	**Other (Various)**
Australia 1 ^22^								
Australia 2 ^23^								
Australia 3 ^21^								
Belgium 1 ^24^								
Canada 1 ^26^								
Canada 2 ^25^								
Canada 3 ^27^								
Chile 1 ^28^								
Columbia 1 ^29^								
Denmark 1 ^30^								
European Union 1 ^60^								
European Union 2 ^59^								
Finland 1 ^31^								
France 1 ^32^								
France 2 ^33^								
Hungary 1 ^34^								
International 1 ^61^								
Italy 1 ^35^								
Ireland 1 ^36^								
New Zealand 1 ^37^								
New Zealand 2 ^38^								
New Zealand 3 ^39^								
Norway 1 ^40^								
Norway 2 ^41^								
Portugal 1 ^42^								
Republic of Korea 1 ^43^								
Spain 1 ^44^								
Switzerland 1 ^45^								
Switzerland 2 ^46^								
Turkey 1 ^47^								
United Kingdom (England) 1 ^48^								
United Kingdom (England) 2 ^50^								
United Kingdom (England) 3 ^49^								
United Kingdom (Scotland) 1 ^52^								
United Kingdom (Scotland) 2 ^51^								
United Kingdom (Wales) 1 ^53^								
United States of America 1 ^58^								
United States of America 2 ^56^								
United States of America 3 ^57^								
United States of America 4 ^55^								
United States of America 5 ^54^								

Abbreviation: MSK, musculoskeletal. Note: Green = Primary condition targeted in document; Yellow = Condition is addressed as a major theme in document; Orange = Condition mentioned as a minor or incidental target in document; Red: Condition not addressed in document.

 Although the documents varied in form and even target condition, there was a commonality in purpose across most documents to improve understanding and awareness of the conditions they addressed and reduce disease burden.

###  Addressing Musculoskeletal Conditions

 A range of MSK conditions were addressed in policies, while very few documents addressed MSK conditions in their full breadth (Table 3). Only 9 documents (European Union 1, International 1, New Zealand 3, Switzerland 2, Turkey 1, the United Kingdom [Scotland] 1, and the United Kingdom [England] 1, 2, and 3) aimed to address MSK health as its primary goal.^39,46-50,52,60,61^ Of those documents in the preceding list, only 3 were comprehensive national MSK policies addressing all parts of the health system (Switzerland 2, Turkey 1 and the United Kingdom (England) 3).^46,47,49^

**Table 3:** Policy Categories, Sub-themes and Transferable Principles Found in Musculoskeletal Policy Documents

**Policy Categories**	**Example Excerpts**	**Identified Sub-themes**	**Transferable Principles**
Service Delivery			
	*“Define specific assistance paths with social integration, health and involvement of social services to support interventions for vulnerable people and/or those in difficult social contexts” *(Italy 1). *“Promote the early initiation of lifestyle modifications to reduce risk, injury, and pain by using effective nonpharmacological approaches and proven self-management education and physical activity programs”* (United States of America 3). *“Once screening is completed, a Healthy Lifestyle and Behaviours Plan will be implemented that is tailored to the individual’s needs. Health providers will advise which exercise is needed as part of this plan, as well as drug management/medication and nutrition management” *(Republic of Korea 1). *“The atlas shows great geographical variation in the treatment of musculoskeletal disease. The information in the atlas provides the professional community with knowledge that can help reduce unfounded variety and secure equivalent health services”* (Norway 2). “*In order to reach people in vulnerable situations more effectively, projects are to be tailored to the needs of the respective target groups and made easily accessible.”* *“The MoC should clearly define the target population and identify any specific priority groups*” (International 1). *“Develop best practice guidelines for rehabilitation in the health system”* (Chile 1). *“To promote an effective, evidence-based, and timely approach to managing musculoskeletal pain and disability that applies a biopsychosocial treatment model to restore workplace function and prevent or minimize work disability as early as possible”* (United States of America 1). *“Work collaboratively to influence commissioners and providers to deliver evidence-based interventions”* (United Kingdom [England] 3). *“Goal 1: Risk factors and Prevention Strategies. Goal 2: Early Diagnosis, Early Treatment and Follow up of Diseases”* (Turkey 1). *“Identify risk factors for severity or impact and use tools where they exist to analyse and stratify risk of progression to long term pain and disability”* (United Kingdom [England] 2). *“All relevant health professionals must be able to perform a valid, standardized, age appropriate musculoskeletal screening assessment”* (Canada 2). *“We have commissioned a research partner to answer which models of MSK and OH service provision are available, where the gaps exist in provision – both geographically and by service type” *(United Kingdom [England]) 3). *“Improved consumer experiences relating to services and pathways measured through: Satisfaction with both access to, and quality of, healthcare services; improved health literacy; consistency of service quality and access across sites”* (New Zealand 1)	Sub-theme 1: Person-centred care	MSK requires care that is respectful of, and responsive to, the preferences needs and values of the individual.
		Sub-theme 2: Identifying and supporting vulnerable and priority populations	Discrimination (overt and institutional) impacts MSK healthcare. Equity should underpin strategy by identifying and targeting services for priority populations. Naming these populations and needs can help prevents such populations falling through the cracks.
		Sub-theme 3: Lifestyle interventions, prevention, early intervention	Complete service packages for MSK health include prevention, early intervention, active and passive treatment, management, recovery, rehabilitation and palliation.
		Sub-theme 4: Interdisciplinary and integrated services	MSK health service delivery is often multidisciplinary and needs to transition to fully integrated or interprofessional care.
		Sub-theme 5: Evidence-based care	Service models should align with best practice evidence and be reviewed regularly. Interventions should be aligned with evidence and reflect the needs of the individual. Low benefit, unnecessary and harmful interventions (low-value care) should be reduced in favour of high value care.
		Sub-theme 6: Access to specialist and rehabilitation services	Health services should be adequately resourced and structured to support rapid access to affordable specialist care for those who need it.
		Sub-theme 7: Risk screening and prioritisation	Prevention and early intervention requires the establishment of systems to identify modifiable risk factors and should target various settings (work and community).
		Sub-theme 8: Service mapping	Ensure access to care by maintaining data on service location and availability, and plan services geographically to enable access where it is needed most.
		Sub-theme 9: Quality of Care	Systems and processes must be in place to ensure high standards of evidence-based care are reached and maintained.
Workforce			
	*“The topic of musculoskeletal diseases is an integral part of the university and non-university training of all professionals involved”* (Switzerland 2). *“Develop a recommendation on minimum requirements for learning content for initial training programs for doctors, dentists, health psychologists, nurses and physiotherapists. Train teachers of inclusive education related to pain management. Develop common learning materials and new learning methods (video lectures, online courses). Compulsory elective courses on pain and its treatment will be created in medical faculties” *(Finland 1). “*Engage in effective inter-professional communication and collaboration with clear documentation to optimise the integrated management of conditions”* (United Kingdom [England] 2). “*The health professional should have access to regular sparring with another colleague who has knowledge of and experience with patient-oriented supervised individual physical training”* (Denmark 1). *“Create and disseminate guidelines, handbooks, and other tools targeting health care providers and insurers that support effective and efficient adoption, use, and maintenance of pr oven self-management strategies”* (United States of America 3). *“Building skills and capacity by providing a set of tools, training and learning events to support local awareness and good practice”* (United Kingdom [England] 3). *“Determine the training needs of the personnel involved (health and non-health) also in relation to the topic integrated management, teamwork, the use of new technologies”* (Italy 1). *“Goal 3: Health practitioners are well-informed and skilled on best practice evidence-based care and are supported to deliver this care”* (Australia 3). *“Strengthen the undergraduate/professional entry-level curricula related to arthritis as part of chronic disease prevention and management for all health care providers”* (Canada 2). *“The framework recognises that practitioners will acquire the capabilities through their pre-and post-registration education (at undergraduate and postgraduate levels) and as their learning and professional development progresses”* (United Kingdom [England] 2).	Sub-theme 1: Workforce Networks	Develop and make use of communities of practice, for clinicians, public health officers, administrators, and policymakers. These should be multi-disciplinary. Digital technology can ease the establishment and maintenance of such networks.
		Sub-theme 2: Resources for workforce use in practice	Clinical guidelines; local service directories; decision-making tools and health workforce training and education materials should be developed and implemented in practice.
		Sub-theme 3: Continuous workforce education	Develop and deliver information, training and education for ongoing professional development and upskilling of the workforce in appropriate prevention, management and care.
		Sub-theme 4: Workforce Support Tools and Systems Support	Resources should be in place to support workforce capacity including specified workforce roles for MSK health and care coordination; resources to test application of new models of care; time and financing resources to undertake training.
		Sub-theme 5: Workforce capabilities	A framework of core workforce qualities and capabilities should be based on high levels of training, qualification and registration; and include recognition of specific skills required for MSK health including: multi-disciplinary skills, cultural competencies, system knowledge and team-based care.
		Sub-theme 6: Undergraduate and Post-Graduate Workforce Education	Include MSK competencies within curricula across formal health workforce education and tertiary training facilities.
		Sub-theme 7: Workforce planning	Project MSK workforce needs and determine suitable roles and qualifications.
		Sub-theme 8: Administrative Workforce	MSK policy, strategy and health system reform should be legitimised through the creation of dedicated administrative units and leadership roles and local, district and national levels.
Medicines and Technologies			
	*“In most primary-care settings, the range of available options for chronic pain management is limited, with the most common options being pharmacological management, specialist or surgical referrals, self-care instructions, or additional imaging and diagnostics”* (United States of America 1). *“Review and monitoring of technical guidelines and monitor opioid prescription” *(Portugal 1). *“Promote self-management in a variety of formats ( eg, group classes, home-based instruction, online options, self-directed guides, mobile health technologies using smart phones, and wearable tracking devices); and settings ( eg, the community, work sites, health care system, and home)”* (United States of America 3). *“Shared decision-making tools are available in all health boards providing information on which people living with persistent pain can make informed decisions about their care”* (United Kingdom [Wales] 1). *“Creation of a health education kit distributed to health professionals”* (France 1).	Sub-theme 1: Pharmacologic and biologic interventions	Medicines should be used appropriately, guided by evidence and processes of health technology assessment and management such as evidence review, technology and data infrastructure supported monitoring and health economic evaluation.
		Sub-theme 2: Digital Technologies to support service delivery	Adopt technologies such as telehealth as a means of improving access.
		Sub-theme 3: Place of medicine in multi-disciplinary care	Pharmacologic treatments should be used, where appropriate, in conjunction with other treatments.
		Sub-theme 4: Safe Medicine use: education and knowledge for citizens	Maintain a strong process of education and awareness in the safe use of medications and have processes to monitor medicine use.
		Sub-theme 5: Biomechanical interventions/living aids	Where aligned to evidence, include adaptive devices and biomechanical interventions in the suite of tools and therapies.
		Sub-theme 6: Appropriate use of opioid medicines	A special focus on the use, safety and risks associated with opioid medication is warranted with resources to guide tapering, provide alternative medicines if indicated and reduce risks of overuse/addiction.
Financing			
	*“Through targeted strategic funding, CAN created collaborative multi-disciplinary teams to work on improving the care of Canadians living with arthritis and the Canadian economy” *(Canada 2). *“The contract for arranging special medical care must agree on eg, the division of labor and the coordination of the activities of the associations of municipalities in the hospital districts under special responsibility. The division of labor must ensure that, in accordance with the organization agreement, the operating unit providing care has sufficient financial and human resources as well as expertise”* (Finland 1). *“Work to provide public and private financing and reimbursement for participation in evidence-based, self-management education and physical activity programs among community”* (United States of America 3). *“To ensure the goals of the NCD strategy can be achieved, financial resources need to be deployed efficiently and in line with the strategic objectives” *(Switzerland 1). *“Research funding and capacity for arthritis and musculoskeletal conditions is commensurate with the burden and cost of these conditions”* (Australia 1). *“In addition to the internal partnerships with other CIHR Institutes, IMHA will work, nationally and internationally, with other funding agencies, and with charities, professional organizations and non-traditional partners in the public and private sector, to address its strategic priorities”* (Canada 1).	Sub-theme 1: MSK targeted funding	Targeted funding for MSK conditions to increase quality and quantity of locally relevant community and health workforce knowledge to address the burden of MSK.
		Sub-theme 2: Funding beyond clinical services	MSK health has a particular connection to multifaceted, long-term and holistic care requiring self-management, community care, physical activity programs, social care and psychological care to be included in health financing and payment models.
		Sub-theme 3: Incentives for coordination, multi-disciplinary and holistic care	Include incentives to ensure citizens receive whole-of-person coordinated services.
		Sub-theme 4: Budget allocation in line with burden	The burden of MSK conditions, defined along multiple lines, should guide the allocation of budget and access to health financing models with a view to return on investment (addressing modifiable risk factors for other NCDs).
		Sub-theme 5: International financing mechanisms	Developing health systems should emphasise MSK health and leverage international partnerships to support reform agendas.
		Sub-theme 6: Affordable services	Essential components of MSK health should be identified and included in payment models and financing systems.
Data and information systems			
	*“Target: the creation of a national data system for Turkey Musculoskeletal System Diseases Prevention and Control Program Evaluation monitoring and reporting” *(Turkey 1). *“Updating of the Information Systems of the Occupational Risk Administrators and Health Promotion Entities, which facilitate the consultation, audit and migration of information, in a timely, efficient, truthful and complete manner” *(Columbia 1). * “Embed data collection into hospital and clinical management systems to capture and analyse treatment and outcomes data to inform clinical decisions and drive quality improvement”* (Australia 1). *“…build databases, in which administrative data converge clinical (health, social-health and social-assistance fields) for evaluation of clinical and organizational results and quality assistance”* (Italy 1). *“Monitor and evaluate their practice and its outcomes, including through data collection and analysis to assure and improve the quality of care, service delivery and address health inequalities”* (Italy 1). *“Increase information sharing, awareness, dissemination, and use of existing and new communication campaigns and tools to reduce OA symptoms and improve OA management”* (United States of America 3). *“Better use of linked data, such as linked primary and secondary care data, linked Census and secondary care data, could allow further investigation into the outcomes for people with MSK conditions”* (United Kingdom [England] 3). *“Justification for the target group should be informed by local data that demonstrate where services are needed the most”* (International 1). *“Coordinated and integrated surveillance systems at multiple levels of the health care systems can promote and enhance quality of care delivered for people living with arthritis”* (Canada 2).	Sub-theme 1: Determining quality indicators	Quality health system performance measures should be developed with aligned with robust monitoring systems.
		Sub-theme 2: Mainstreaming monitoring and evaluation	Make full use of pilot monitoring and evaluation programs, rollout and scale up to build the evidence base for MSK health system improvement locally.
		Sub-theme 3: Data systems infrastructure	Systems of routine data collection should be implemented, data collected, collated and reported to support monitoring needs and progress against quality markers. Systems can be built on existing administrative processes or through the deployment of surveys. Local data should be collated in national systems.
		Sub-theme 4: Data reporting, dissemination and use	Build appropriate data collection, monitoring and evaluation and piloting systems to underpin decisions in 'learning health systems' (See also governance sub-theme 4).
		Sub-theme 5: Linking local data sources	Clinical digital health informatics platforms should enable connected care and monitoring across the health system in primary, secondary, tertiary, community, social care settings.
Leadership and governance			
	*“Establish and fund a National Arthritis Collaboration to engage with and align efforts across multiple stakeholders, sectors and levels of the health system to drive improvements in arthritis prevention and management” *(Australia 1). *“Working groups will be formed according to program objectives, in which the members of the general assembly take part in accordance with their job descriptions. Each working group prepares proposals for the planning, execution, evaluation and development of the work in its field specified in the action plans, submits it to the executive board, and carries out the approved activities*” (Turkey 1). *“Objective 1.2 Integrate workers' health protection measures into economic development policies and poverty reduction strategies” *(Columbia 1). *“The Arthritis Alliance of Canada, a coalition of arthritis organizations from across the country, has developed Joint Action on Arthritis: A Framework to Improve Arthritis Prevention and Care in Canada to facilitate collaboration on effective solutions and to secure the collective leadership commitments to make change happen” *(Canada 2). *“GPs also play an essential clinical leadership role in multi-professional teams, employing a growing range of clinicians in their services and commissioning MSK services for their local populations” *(United Kingdom [England] 2). *“Local clinical and administrative champions who are supported by their organisation to adopt a leadership position in the development, communication and implementation of the Model of Care” *(Italy 1). *“Strengthening political leadership to ensure a coordinated and intersectoral approach, and integration of health issues into all policies is of paramount importance” *(International 1). *“The Alliance and its members have taken a leadership role in developing this Framework and will be approaching governments and other arthritis stakeholder organizations with specific requests for support and participation in implementing its initial priorities and actions”* (Canada 2). *“It is recommended that a uniform registration takes place across the municipalities through reporting to national databases to the extent that they exist”* (Denmark 1). *“The champions tasked with spearheading the MoC should be up-skilled in implementation science or change leadership in order to act as effective change agents”* (International 1).	Sub-theme 1: Championing MSK health.	The roles, knowledge and capacity of advocates should be acknowledged and harnessed for action.
		Sub-theme 2: Establishing systems for decision making	Establish responsible committee structures for decision-making in MSK health.
		Sub-theme 3: Delegating leadership	Knowledge, responsibility, and authority may be located across levels of government, in multiple parts of the health system and non-governmental organisations requiring delegation, multi-stakeholder governance and coordination.
		Sub-theme 4: Data for leadership	Build data collection, monitoring and evaluation and piloting systems to underpin decisions in 'learning health systems.'
		Sub-theme 5: Building local capacity and capability in leadership	The capacity and capability to undertake responsibilities and perform functions of leadership and governance should be supported across the system.
Citizens, consumers and communities			
	*“The focus is on people: The focus is on people. It does not matter whether it is a healthy person, a person with risk factors for a musculoskeletal disorder or a person who is affected by a musculoskeletal disorder” *(Switzerland 2). *“Every Canadian with arthritis must have access to accurate information and education on arthritis that meet a defined set of criteria and are appropriate to their age and stage of disease”* (Canada 2). *“State and professional organizations should increase consumers,’ primary care providers,’ and medical specialists’ awareness of, and access to, function- and work-oriented pain management services that use a biopsychosocial approach, especially patient education and counselling, emphasizing the health benefits of continued employment for most patients” *(United States of America 1). *“The programme provides education on pain and lifestyle, physical exercise training and a range of techniques to assist the individual's management of pain conditions” *(Switzerland 1). *“Goal 2: Consumers, their carers and the wider community are more empowered knowledgeable and supported to understand and manage pain” *(Australia 3). *“Identify and engage community organizations ( eg, faith-based and cultural or ethnic groups; neighborhood associations; senior centers; and parks, recreation, fitness, health, and wellness organizations) that may not be aware of or using the effective intervention strategies or offering evidence-based programs to help with OA” *(United States of America 3). *“Prepare material to raise awareness and sensitize, for managers, professionals and patients about the importance of adequate pain care” *(European Union 1).	Sub-theme 1: Public education and awareness	Increase community awareness and understanding of the MSK conditions, their (modifiable) risk factors, prevention and management including role of self-management.
		Sub-theme 2: Working in partnership	Utilise partnerships with consumers and other organisations and associations to advance MSK health agendas.
		Sub-theme 3: Identifying and supporting vulnerable and priority populations (cross-over with service delivery sub-theme 2)	Equity should underpin strategy by identifying and including engagement of specific priority populations in inclusive and accessible ways to prevent discrimination and support culturally-appropriate care and care equity.
		Sub-theme 4: Citizen science and data	Technologies can provide opportunities to make use of citizen and consumer-level data capture to guide self-management, support shared decision making and promote population-level monitoring.
Research and innovation			
	*“In the “Research” area, the goal is to improve the data on musculoskeletal diseases. In addition to adapting or expanding existing health statistics and registers. Research funding also contributes to this. Innovative projects to care for people with musculoskeletal diseases should increasingly be scientifically supported” *(Switzerland 2). *“This entails building and sustaining research networks that aim to advance research, training and knowledge translation across multiple disciplines, with a focus on expediting knowledge uptake to mobilize and build IMHA’s research community”* (Canada 1). *“Facilitate the formation of multi-disciplinary research groups to address knowledge gaps in arthritis prevention, management and models of care”* (Canada 1). *“Provide dedicated funding for musculoskeletal research fellowships for clinician researchers and researchers at all career stages”* (Australia 1).	Sub-theme 1: Investment in research	Invest in MSK health research across the spectrum including from early detection, prevention, diagnosis, management, treatment, rehabilitation and recovery.
		Sub-theme 2: Research dissemination, translation, and implementation	Translation and implementation strategies should be required to accompany application of new research to support whole-of-system reform.
		Sub-theme 3: Knowledge, skills and capacity to undertake research	Research and innovation should be approached in conjunction with and serve to benefit the MSK health workforce and expand research capacity in the MSK health workforce.
		Sub-theme 4: Research policy and funding systems	Develop high-level strategy and coordination of research for MSK health; with systematic identification of research priorities and allocated funding.

Abbreviations: MSK, musculoskeletal; NCDs, non-communicable diseases; MoC, model of care; OA, osteoarthritis; IMHA, Institute of Musculoskeletal Health and Arthritis.

 In our pool, six documents were broad national health plans or national NCD plan with substantial component on addressing MSK health (Italy 1, Finland 1, Hungary 1, Republic of Korea 1, Norway 2, and Switzerland 1).^31,34,35,41,43,45^ Other documents were either cross-jurisdictional, or only addressed parts of the health system (eg, residential care, or allied health workforce). Three countries (Australia, the United States, Canada) appeared to have a series of policies that each addressed different MSK conditions, which combined could be interpreted as entailing a comprehensive MSK policy response.

 The most commonly addressed MSK conditions across all policies were pain (general, 19 policies), osteoarthritis (13 policies) occupational health (12 policies) and low back pain (12 policies) (Table 2). The MSK strategy of Turkey^47^ contained a substantial focus on different conditions, compared with all other policy documents. While it did address low back pain, osteoarthritis and inflammatory rheumatic conditions, it also dedicated equal specific attention to familial Mediterranean fever and Behçet’s disease, indicating the country-specific burden of those conditions.

###  Policy Themes and Principles

####  Targets and Goals

 Despite the variety in document formats and target conditions, there was a surprising consistency in the themes present in policy targets and goals. Almost all policies aimed to reduce the target burden of the diseases, frequently citing the relatively little attention given to MSK conditions in the health system compared to the known burden. Other key themes are listed in Box 1.

**Box 1. Common Themes Found in Targets and Goals in MSK Policy Documents**

**1. Reduce burden **

Measuring and reducing number of people living with MSK conditions.

**2. Improve access to services **

Understanding service availability and moving towards an increased number of citizens that can access MSK services when they are needed.

**3. Improve patient outcomes **

Understanding and improving MSK health following diagnosis.

**4. Knowledge, awareness and outcomes **

Improving knowledge of MSK conditions, how they can be prevented and when to take action among the general population as well as health care providers.

**5. Improve quality of care **

Understanding and improving the quality of MSK services.

**6. Target priority populations **

Understanding who is at risk of MSK disorders and establishing systems to reach those populations.

** 7. Research and investment**

Funding to improve knowledge and understanding of MSK prevention and treatment.

**8. Cost-efficiency **

Calculating the broader costs of MSK disorders and the benefits of early detection and treatment.

-----------------

Abbreviation: MSK, musculoskeletal.

####  Policy Content

 Across the 8 policy categories, we inductively derived 47 sub-themes. Table 3 summarises the strategies outlined across policies, presenting the derived sub-themes by analytic category with exemplar excerpts as evidence, along with the transferable derived principles.

 The most comprehensively addressed major category across the policy documents was MSK health services. This included the range and types of services needed to promote MSK health, and how these services could be delivered. Many documents included texts that emphasized MSK health impairment as a collection of conditions that impact health and wellbeing over long periods of life and requires multi-disciplinary, coordinated services, individual-level assessment and tailored treatment. We drew a total of 9 sub-themes and transferable principles from the documents as they relate to service delivery.

 The second most broadly addressed major category in the documents was centred around the health workforce. This is unsurprising considering the close connection between health services and human resources required for care delivery. Furthermore, with MSK being one of the most common reasons people seek primary care, and the recognized lack of primary care availability in many parts of the world by their own governments, particularly in underserved communities, this makes sense to be one of the more common themes. The need for formal and continuing education, as well as workforce planning and support tools were the most frequent sub-themes. In total, we found 8 distinct sub-themes and transferable principles with respect to the MSK health workforce.

 The next two most broadly raised categories in the texts related to “financing” and “medicines and technologies.” The place of pharmacological and biological treatment in multidisciplinary care, medical education and knowledge for citizens; and the appropriate use, in particular concerning opioid medications, dominated texts around medicines. We determined 6 distinct sub-themes concerning medicines and technologies. Given the prevalence of texts around the appropriate use of opioid medication, we determined this warranted a separate distinct sub-theme.

 There were very few documents that covered in-depth how MSK services, models of care or other activities addressing MSK health should be financed. We relied on smaller text excerpts from the breadth of documents to draw 6 distinct sub-themes and abstracted principles related to this category. Where financing was discussed, texts focused on ensuring that systems of risk pooling, public funding, safety nets, and reducing out-of-pocket costs should be in place for enabling affordable access to health services.

 Almost all documents contained policy statements concerning data monitoring and information systems; with a considerable number of common themes and underlying principles. Ensuring that the quality of services and outcomes are measured, and building systems to collate data through standardized processes were key features in most policies. We determined 5 distinct sub-themes and transferable principles related to data and monitoring.

 We also found 5 sub-themes in texts related to leadership and governance, although this was not commonly addressed specifically as an area for action. Rather we found a reference to the roles and responsibilities allocated to organisations and individuals within the system, as well as the decision-making structures that have been, or will be, established to govern MSK health. We also found a single reference to the roles that non-governmental organisations play in MSK health, particularly in agenda-setting and as issue-area champions.

 Finally, we drew 4 distinct sub-themes in each of the inductively coded analysis categories. With respect to citizens, consumers and communities, most policies contained strategies or goals to educate and empower citizens with better knowledge of MSK health for both prevention and treatment. Policies also focused on reaching priority populations, either through screening, planning or by developing culturally sensitive models of care.

 Policy documents that were comprehensive strategies targeting whole-of-systems approaches included calls for increased research and innovation for improving MSK health. This included strategies to ensure investment in research was commensurate with the burden and setting research priorities; for example, by means of establishing high-level research systems and funding mechanisms. Notably, only a minority of policies addressed a life course approach to MSK health or mentioned MSK health in children or younger people. Polices Canada, the United Kingdom, Australia, and France discussed childhood conditions including juvenile arthritis, bone health and pain care, while a life course approach was recommended in policies from Canada, Switzerland, and Australia.

## Discussion

 Our policy content analysis has found a paucity of policy documents that address the breadth of MSK conditions under one overarching strategy. Critically, very few documents brought together all major policy themes including the WHO Health System Building Blocks, as well as policy themes related to ‘research and innovation,’ and ‘citizens, consumers and communities.’ These findings underline the importance of creating tools and resources to facilitate MSK policy learning and sharing from the breadth of many policy examples, rather than the depth of a few.

 In the few countries where a system-wide overarching policy and strategy to guide MSK health has been developed, such as England, Turkey, and Switzerland, this is likely to have been the product of particular policy entrepreneurship and a facilitating policy context. We propose further research into the history of the development of these policies to determine facilitating factors would be important to understand historical context and to inform prospective policy evolution and evaluation.^6,62^ This approach has been used to better understand policy and global priority evolution related to NCDs generally.^63^ However, even among these few national-level strategies, the variation in format and nature and depth of content, indicates that we are still firmly in a phase of sharing policy experiences and tools, with a focus on engaging in policy learning, rather than supporting widespread implementation and monitoring. Global technical advice to support the establishment of MSK policy has been lacking but could contribute to increased efficiency in the development of MSK policies at the national level, and indeed, an empirically-derived strategic framework has been developed recently for this purpose, as well as more nuanced and context-specific recommendations for low- and middle-income settings.^2,17,18^

 There are opportunities for policy entrepreneurs, from government, industry or civil society sectors to draw on the principles itemized in this review to drive MSK policy evolution locally. This is especially the case in countries that have already invested in monitoring and recording of MSK burden – and we found many examples of this as an incidental finding in our document collection – as well as countries where population health monitoring of MSK health is absent or emerging.^64^ Indeed, as the burden attributed to MSK health impairment becomes more widely recognised and countries move towards arresting this burden, as recommended by Global Burden of Disease findings,^4,14,65^ there will be an increasing need to identify extant and transferable policy solutions and foci. These data will be relevant not only to individual nations but also global health agencies such as the WHO, where programmatic activity related to MSK health is emerging. The combination of the evidence of burden, with examples of how strategies can be formulated and implemented can be used to pry open the policy window. This has become even more relevant due to both direct and indirect effects of the coronavirus disease 2019 (COVID-19) pandemic on MSK health – from the complex interlinkages between COVID-19, social circumstances and MSK pain; through the changing policy focus on local and global health authorities. With the COVID-19 pandemic drawing policy attention away from chronic conditions, including MSK health over the past 3 years, strong evidence and feasible policy solution are even more important tools in the tightened space of agenda setting.^66^

 Patterns and trends in the documents we analysed indicate that there may be several pathways that can be taken in the development of MSK policy at a country level.

 For example, several countries (for example the United States and Australia) have built a series of policies targeting MSK specific conditions, rather than a single overarching MSK strategy. This may be a more feasible approach to developing MSK policy than a single strategy in some settings; while in others an integrated single strategy may be more resource efficient.^67^

 Another example is dispersing responsibility and authority for policy development. In some countries, notably Australia and, Canada, MSK policies were also authored by non-government organizations (including consumer advocacy and professional societies) and research institute with expertise in specific MSK conditions; with a mix of funding, endorsement or co-production with government. The relationship between peak bodies, national research institutes and governments differs from country to country but could also provide important resources and capacity in the development of new policy and system strategies.

 As mentioned above, based on our operational definition, we identified a far greater number of clinical guidelines for MSK health, or reports on MSK disease burden, than we did policy documents. This is despite our search not targeting this type of document specifically. Clinical guidelines may be a useful foundation for the further development of MSK policy in more countries around the world, including LMICs, as it signals the challenges and size of evidence-practice gaps at service and clinical levels. Measuring and understanding the burden of disease and having sound best practice person-centred care are the foundations of understanding service and workforce needs and building the resources for system level strategies and MSK models of care.^68^

 This finding likely reflects an existing strong research network around disease surveillance and monitoring, including the MSK burden and the ensuing strong evidence base.^4^ Importantly, similar burden of disease documents were also identified from LMICs, potentially pointing to emergent activity for health systems strengthening in these countries, despite a recognised lack of local MSK population health surveillance.^64,69^ Most documents were also dominated by goals, targets, service provision and strengthening the workforce, with considerably less focus on governance, technologies and workforce and only a few documents included aspects of monitoring, innovation and community engagement. Apart from a few, we also noted that most policies did not frame discussions around a life course approach or address MSK health in children. This is unusual for policies addressing an NCD where a life course approach is typically a guiding principle, the strong association between disease expression and life course behaviours, and that MSK impairments are common in children. Across all major themes, there is considerable potential for further policy learning and related system strengthening contextualised for country-specific purposes.

 While we identified many national clinical guidelines and reports of national MSK disease burden, there were few system-level policies, strategies or action plans. This suggests that while there has been positive progress in recognizing burden of disease and articulating approaches for clinical management, system-level strengthening is rarely purposively and strategically developed in current policy. We found only 3 documents that could be considered system-wide strategy or policy for MSK health conditions at the national level^46,47,49^ and two at the international level.^59,61^

 Collectively, the pool of documents contained a breadth of key issues, themes and principles. The content analysis of the policies triangulated closely with independently collected data from aligned mixed-methods research in our broader program of work on empirically deriving a co-designed health systems strengthening response for MSK health.^16,17^ This close alignment between independently collected and analysed data validates the concurrent and construct validity of our findings. Importantly, the priorities and strategies articulated by key informants and the global MSK community sampled in our aligned work^18^ are also reflected closely in national policy documents, suggesting an evolution of policy that is reflective of community expectations.

###  Strengths and Limitations

 This comparative policy analysis was the first to provide a comprehensive global search through multiple data collection steps, including networks facilitating the collection of documents in multiple languages combined with a global. Our refinement of categories enabled comparisons across a wide range of document types; enabling collation, analysis and extraction of principles in a policy field where complete, comprehensive national strategies were almost non-existent. The use of interpretive analysis methods enabled the extraction of principles that can be interpreted and applied across contexts, while maintaining the policy learning from original local country-level experience. This work was also strengthened by parallel work undertaken within a wider project that included eDelphi expert panel views on key themes and principles thereby enabling concurrent validation.^17^

 We excluded documents addressing road traffic injury and injury through violent trauma and acknowledge these policy areas as critical for MSK health. We only included policy documents addressing NCDs if there was explicit and substantial inclusion of MSK policy, strategy, or targets, and acknowledge the close interplay between other NCDs and MSK health, and the common shared risk factors.

 While our initial web-based search focused on the 30 most populous countries; and was then supplemented by an expert round and responses to an e-Delphi with participants from 72 countries, it is possible that our search failed to uncover all relevant documents, particularly those from less populated countries.

## Conclusion

 The development and use of national policies for MSK health is still in its infancy. Very few national policies covered multiple MSK conditions; nor did many documents in our analysis cover all health system building blocks or supplementary categories of MSK policy interest. However, the collective pool of documents contained a breadth of key issues, themes and principles and collated and grouped this content covered key principles and abstract key principles that can helpfully guide policy sharing and learning. Future global guidance to assist countries to develop their own MSK policies and strategies can draw on implemented principles across the health system building blocks, including service delivery, workforce, medicines and technologies, financing, data and information systems, and governance. Existing policy also embody underlying principles of action with respect to citizens, consumers and communities including public education and awareness and working in partnerships.

 These collective key issues, themes and principles may be helpful in informing the development of a global policy and technical guidance on action to improve MSK health that is grounded in local experiences.

## Ethical issues

 This article reports on documentary analysis as well as secondary data analysis of an existing survey dataset for which Human Research Ethics Committee approval had been granted by Curtin University, Australia, in 2020 (HRE2020- 0183).

## Competing interests

 Authors declare that they have no competing interests.

## Authors’ contributions

 CHS and AMB conceptualised the study. AMB, DKG, JJY, SS, CHS, and SJ developed and performed search strategies. CHS and SP undertook data extraction and analysis and AMB and JJY performed verifications. AMB, DKG, HS, and LM contributed additional refinement of definitions and inclusions/exclusions. All authors reviewed the results and participated in analytical cycles. CHS lead the writing of the manuscript together with SP and AMB. All authors contributed to the manuscript including final review.

## Funding

 This project was funded by a grant awarded by the Bone and Joint Decade Foundation with additional funding provided by Curtin University, Australia, and guided by an international external steering group of experts in the field of musculoskeletal health.

## Supplementary files

Supplementary file 1. Search Strategy.
