# Supplementary material for: From Local Action to Global Policy: A Comparative Policy Content Analysis of National Policies to Address Musculoskeletal Health to Inform Global Policy Development
Source: Int J Health Policy Manag. 2023 Jan 4;12:7031. doi: 10.34172/ijhpm.2022.7031 (PMC10125103; doi:10.34172/ijhpm.2022.7031)
Supplement: Supplementary file 1 — Search Strategy. [file ijhpm-12-7031-s001.pdf]

**Article title:** From Local Action to Global Policy: A Comparative Policy Content Analysis of National Policies to Address Musculoskeletal Health to Inform Global Policy Development

**Journal name:** International Journal of Health Policy and Management (IJHPM)

**Authors' information**: Carmen Huckel Schneider[1]*, Sarika Parambath[1], James J. Young[2,3], Swatee Jain[4], Helen Slater[5], Saurab Sharma[6,7], Deborah Kopansky-Giles[3,8], Lyn March[9], Andrew M. Briggs[5]

[1]Menzies Centre for Health Policy and Economics, Faculty of Medicine and Health, University of Sydney, Sydney, NSW, Australia.

[2]Center for Muscle and Joint Health, Faculty of Health Sciences, University of Southern Denmark, Odense, Denmark.

[3]Department of Research, Canadian Memorial Chiropractic College, Toronto, ON, Canada.

[4]Sydney Musculoskeletal, Bone & Joint Health Alliance, Faculty of Medicine and Health, University of Sydney, Sydney, NSW, Australia.

[5]Curtin School of Allied Health, and Curtin enAble Institute, Faculty of Health Sciences, Curtin University, Perth, WA, Australia.

[6]School of Health Sciences, Faculty of Medicine and Health, University of New South Wales, Sydney, NSW, Australia.

[7]Centre for Pain IMPACT, Neuroscience Research Australia, Sydney, NSW, Australia.

[8]Department of Family & Community Medicine, University of Toronto, Toronto, ON, Canada.

[9]Florance and Cope Professorial Department of Rheumatology, Royal North Shore Hospital and Kolling Institute, University of Sydney, Sydney, NSW, Australia.

(Corresponding author: carmen.huckelschneider@sydney.edu.au)

**Supplementary file 1.** Search Strategy

A systematic search for policies in the most 30 most populated countries was performed using Google from 01 July 2020 to 15 Aug 2020 for each country. Countries were identified by population size using UN World Population Prospects. Search terms for musculoskeletal condition descriptors used to identify organisations [See Box 1 below] were supplemented with the search terms [“policy OR strategy OR action plan OR strategic framework OR health indicators”] along with a country name to locate potential national policy documents.

**Box 1: Search terms of musculoskeletal condition descriptors used to identify organisations**

**Rheumatology**

Rheumatology+Association+Society + Country name
Rheumatism+ Association+Society + Country name
Arthritis+ Association+Society + Country name
Musculoskeletal+ Association+Society + Country name
All of the above + region name + any city name (when country-specific not identified)
**International organisation site: International League of Associations of Rheumatology**
http://www.ilar.org/partners/

**Orthopaedics**

Orthopaedics+Association+Society + Country name
Orthopaedics Specialist+ Association+Society + Country name
Orthopaedics Department+ Hospital+ Society + Country Name
Orthopaedics + Publications+ Country name - Searching author names from respective country
All of the above + region name + any city name (when country-specific not identified)
**International organisation site: SICOT (Société Internationale de Chirurgie Orthopédique et de Traumatologie)** https://www.sicot.org/about-sicot

**Pain Medicine**

Pain+ Association+Society + Country name
Pain + Chapters+ IASP+ Country name
Anaesthesiology+ Pain+ country when no other contact of pain identified
All of the above + region name + any city name (when country-specific not identified)
**International organisation site: International Association for the Study of Pain**
https://www.iasp-pain.org/

**Paediatrics/Adolescent health**

Paediatrics+ Association+ Country name
Paediatrics+ Society+ Country name
Paediatric+ Country name+ publications - Searching author names from respective country
All of the above + region name + any city name (when country-specific not identified)
**International organisation site: International Paediatric Association** https://ipa-world.org/page.php?id=326

**Rehabilitation**

Rehab+ Rehabilitation+ Association+ Country name

Rehab+ Rehabilitation + Country + Publication
All of the above + region name (when country-specific not identified)

**Gerontology/Geriatrics**
Geriatrics+ Society+ Country name
Gerontology+ Association+ Society+ Country name
Geriatrics+ Publication+ Country name
All of the above + region name (when country-specific not identified)

**Physiotherapy**
Physio+ association + Society+Country name
Physical Therapy+ Society + Country name
Physiotherapy+ Association+ Country name
All of the above + region name (when country-specific not identified)
**International organisation site: World Physiotherapy** https://world.physio/

**Chiropractic**
Chiropractor+ society + Country name
Region name + Chiropractor+ Association
Capital city name+ Chiropractor+ Association
**International organisation site: International Chiropractor Association**
https://www.chiropractic.org/about/
**International organisation site: Chiropractic Diplomatic Corp**
https://www.chiropracticdiplomatic.com/?s=ethiopia

Often, a combination of these search terms would lead to the webpage of a government agency or department relevant to MSK health or chronic pain, where hand searching of the entire website for relevant documents was performed. In addition, hand searching of other linked government agencies webpages was also performed. Finally, reference lists of retrieved policy documents were hand searched for additional documents relevant to MSK health and chronic pain policy.
